# Lhermitte’s Phenomenon as the Presenting Symptom of Vitamin B12 Deficiency With Complete Reversibility of Neurological and Radiological Abnormalities

**DOI:** 10.7759/cureus.95922

**Published:** 2025-11-01

**Authors:** Hadiza Ibrahim, Mira Ibrahim, Hadaya Aldhaheri, Mahfoud Elbashari, Hesham Eissa, Amani Alzaabi

**Affiliations:** 1 Internal Medicine, Zayed Military Hospital, Abu Dhabi, ARE; 2 Neurology - Internal Medicine, Zayed Military Hospital, Abu Dhabi, ARE; 3 Neurology, Zayed Military Hospital, Abu Dhabi, ARE

**Keywords:** cercical myelopathy, lhermitte sign, myelopathy, vitamin b12 deficiency symptoms, vit b12 deficiency

## Abstract

Vitamin B12 deficiency is a potentially reversible cause of neurological dysfunction, including myelopathy. We present a case of a 37-year-old man with Lhermitte’s sign and distal paresthesia but without focal neurological deficits. Laboratory evaluation showed low-normal serum B12 with elevated intrinsic factor antibodies. MRI revealed cervical spinal cord hyperintensity involving the posterior columns (C2 to C5), which fully resolved following vitamin B12 supplementation. This case underscores that neurological manifestations of B12 deficiency may occur even with “normal” B12 levels and no hematologic abnormalities and highlights the importance of early recognition and treatment to reverse both clinical and radiologic findings.

## Introduction

Vitamin B12 (cobalamin) is essential for DNA synthesis, erythropoiesis, and the maintenance of neurological function. Its deficiency can lead to a broad spectrum of hematologic and neuropsychiatric complications, which may occur independently. Neurological manifestations such as paresthesia, gait disturbance, cognitive impairment, and myelopathy often precede anaemia or macrocytosis and may be the sole presenting features in some patients [1,2].

Subacute combined degeneration (SCD) is a well-recognized consequence of B12 deficiency, involving demyelination of the dorsal and lateral columns of the spinal cord, typically affecting the cervical and upper thoracic regions [3]. Magnetic resonance imaging (MRI) serves as a useful adjunct, revealing T2-weighted hyperintensities in the posterior columns that may resolve with treatment [4,5].

Lhermitte’s sign is characterised by a transient electric-shock sensation radiating down the spine and limbs upon neck flexion. It reflects posterior column involvement and is most often associated with demyelinating diseases, though metabolic causes such as B12 deficiency are also implicated [6].

Furthermore, up to 14% of patients with neurologic B12 deficiency may have a normal neurological examination at presentation [1]. Reliance on serum B12 levels alone can be misleading, particularly in borderline or “low-normal” ranges. Functional biomarkers such as methylmalonic acid (MMA) and homocysteine offer improved diagnostic accuracy in these cases [7,8]. This case highlights an unusual presentation of B12 deficiency with Lhermitte’s phenomenon and reversible radiologic abnormalities in a patient without anemia.

## Case presentation

A 37-year-old man with no prior comorbidities presented with a two-week history of electric shock-like sensations radiating down the spine on neck flexion (Lhermitte’s sign), along with distal paresthesia in all four limbs. He denied any weakness, visual changes, sphincter dysfunction, constitutional symptoms, or prior neurological issues.

Neurological examination was normal, including tone, strength, reflexes, coordination, and all sensory modalities. There were no signs of myelopathy, cerebellar involvement, or long tract signs.

Initial laboratory investigations were largely unremarkable (Table 1). Complete blood count, thyroid-stimulating hormone (TSH), and folate levels were within normal limits. Serum vitamin B12 was in the low-normal range, both intrinsic factor and parietal cell antibodies were positive, suggesting underlying pernicious anemia. Peripheral smear revealed macrocytosis but there were no hypersegmented neutrophils. Nerve conduction studies were performed and were normal.

**Table 1:** Summary of Laboratory Investigations There was evidence of macrocytosis, vitamin B12 was in the low-normal range, intrinsic factor and parietal cell antibodies were positive, supporting the diagnosis of functional B12 deficiency. Folate and thyroid function were unremarkable. No evidence of anemia.

Parameter	Result	Reference Range	Interpretation
Hemoglobin	14.7 g/dL	13.0-17.0 g/dL	Normal
Mean corpuscular volume (MCV)	98.6 fL	80-95fL	Normal
Vitamin B12	320 pmol/L	200-950 pmol/L	Low-normal
Folate	12.3 ng/mL	>5 ng/mL (typical)	Normal
Thyroid-stimulating hormone (TSH)	3.2 mIU/L	0.3-4.5 mIU/L (typical)	Normal
Intrinsic factor antibodies	Positive	Negative	Abnormal
Parietal cell antibodies	Positive	Negative	Abnormal

**Figure 1:**
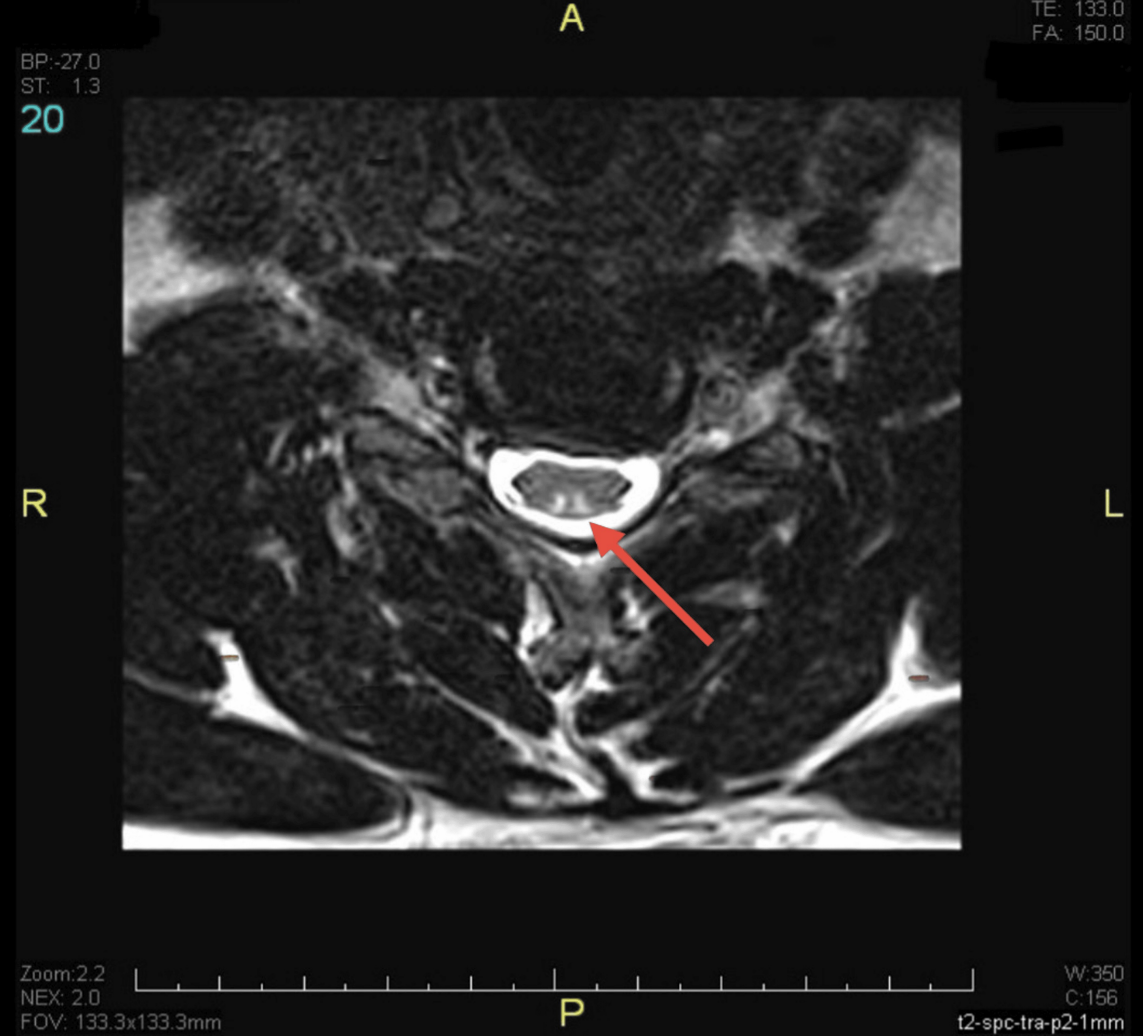
Axial T2-Weighted MRI of Cervical Spine Showing Posterior Column Involvement Axial T2-weighted MRI at the C3 level reveals bilateral symmetrical hyperintensity in the dorsal columns (red arrow), forming the characteristic “inverted V sign” seen in subacute combined degeneration due to vitamin B12 deficiency. The anterior cord is spared.

**Figure 2:**
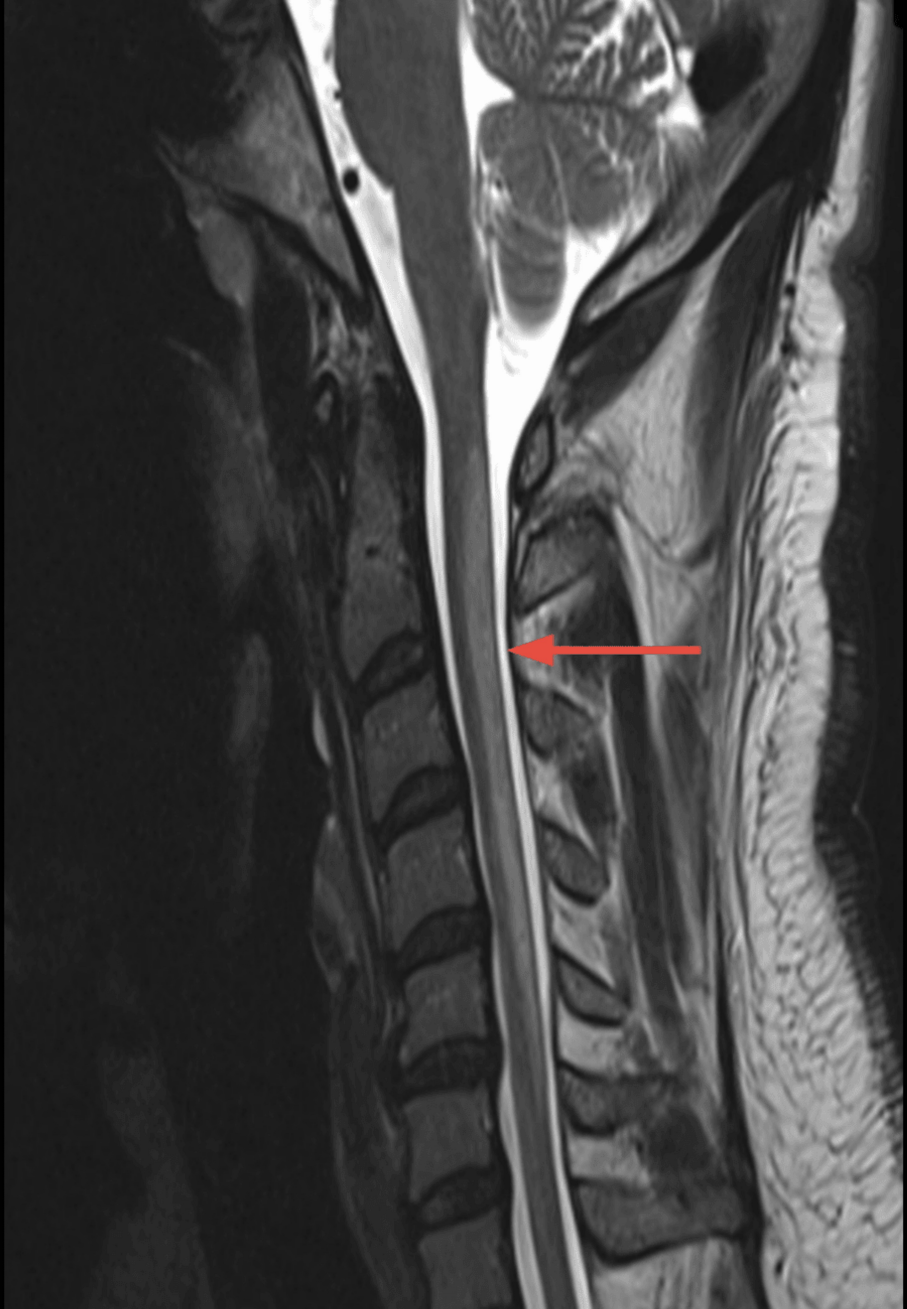
Sagittal T2-Weighted MRI of Cervical Spine Demonstrating Dorsal Column Hyperintensity Sagittal T2-weighted MRI of the cervical spine shows linear bilateral hyperintensity involving the dorsal columns from C2 to C5 (red arrow), without mass effect or contrast enhancement. These findings are characteristic of subacute combined degeneration due to vitamin B12 deficiency. No evidence of cord expansion or compressive lesion is noted.

Magnetic resonance imaging (MRI) of the cervical spine revealed bilateral, symmetric T2 hyperintensity of the dorsal columns extending from C2 to C5, without contrast enhancement or mass effect. These findings were consistent with early SCD of the spinal cord (Figures 1, 2).

**Figure 3:**
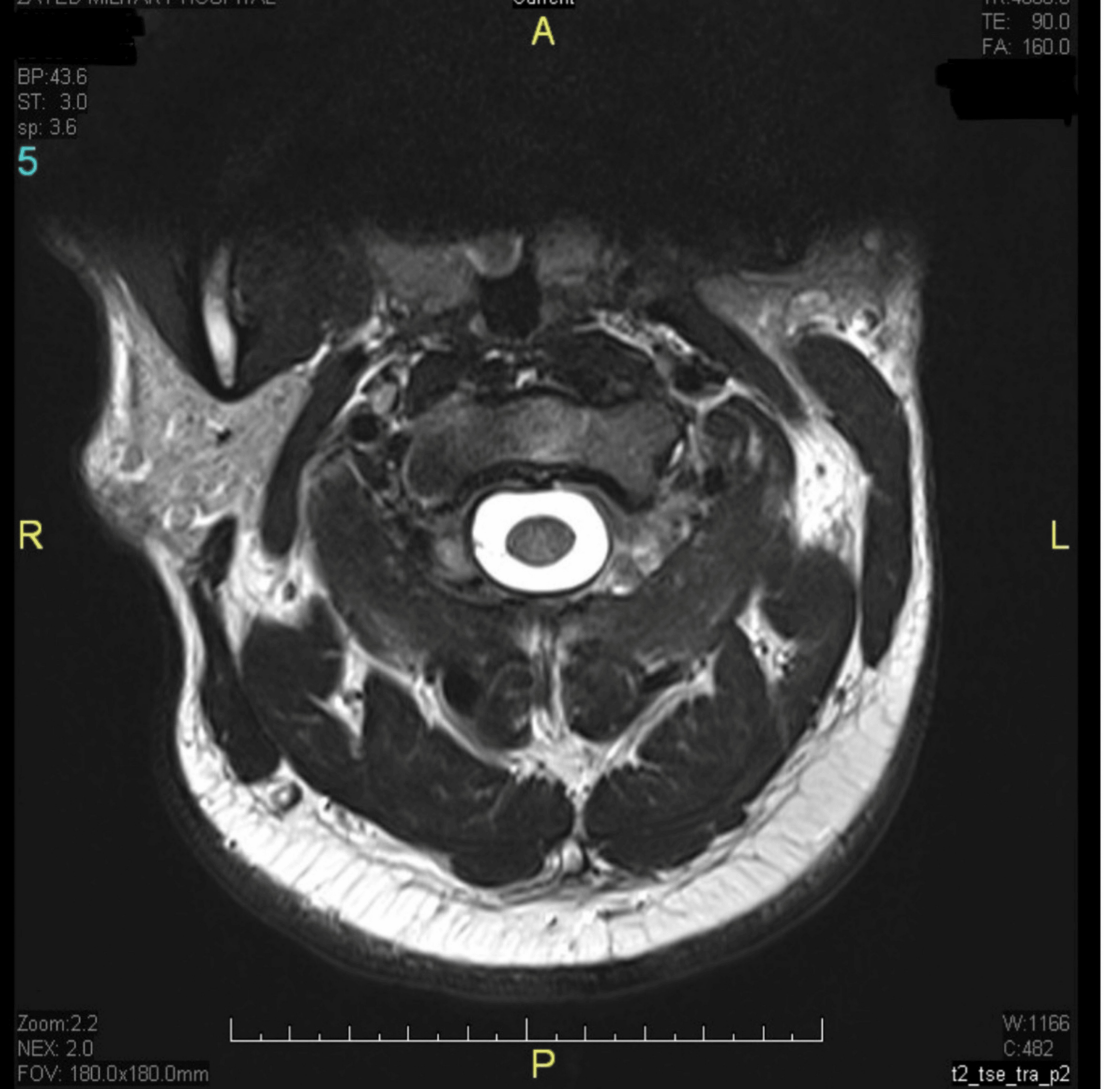
Follow-Up Axial T2-Weighted MRI Demonstrating Resolution of Cord Signal Abnormalities Repeat axial T2-weighted MRI demonstrates resolution of the prior dorsal column hyperintensity (compare with Figure 1). The spinal cord signal has normalized, consistent with radiological recovery after treatment.

**Figure 4:**
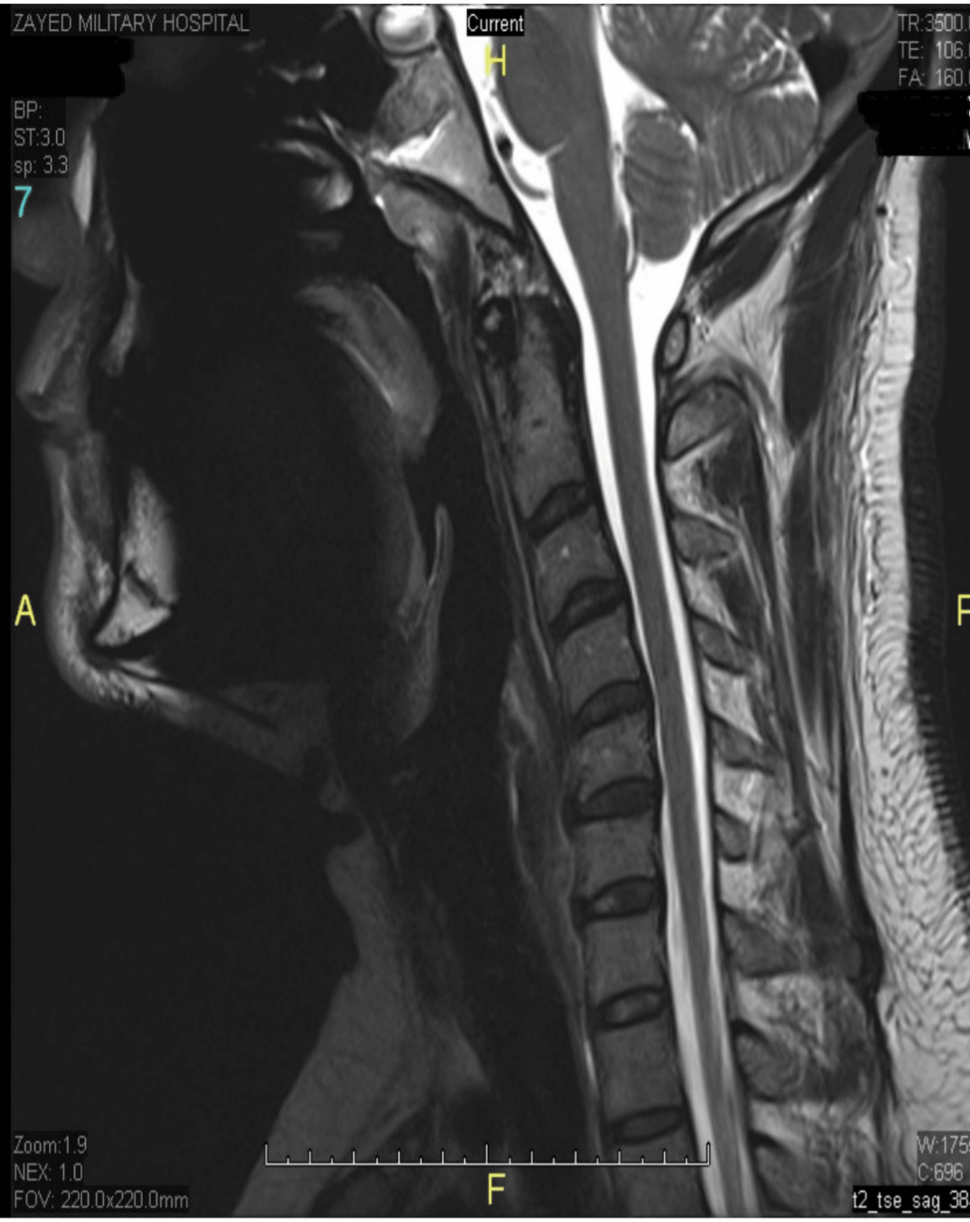
Repeat Sagittal T2-Weighted MRI Demonstrating Resolution of Dorsal Column Hyperintensity Repeat sagittal T2-weighted MRI of the cervical spine shows resolution of the previously noted bilateral dorsal column hyperintensity. No residual signal abnormality is seen, indicating radiological improvement following vitamin B12 replacement.

The patient was initiated on intramuscular hydroxocobalamin 1 mg on alternate days for two weeks, then once weekly. By the third week of treatment, Lhermitte’s sign had resolved completely, and paresthesia had significantly improved. Follow-up serum B12 levels at three months post-treatment showed a significant increase to 450 pg/mL and repeat MRI showed complete resolution of the previous signal changes (Figures 3, 4). He remained asymptomatic with ongoing monthly maintenance therapy.

## Discussion

This case illustrates a rare but instructive presentation of vitamin B12 deficiency, with Lhermitte’s sign as the initial and sole neurological symptom. While typically associated with demyelinating diseases such as multiple sclerosis, Lhermitte’s sign may also reflect reversible dorsal column dysfunction caused by metabolic insults like SCD [4,6]. In such cases, early diagnosis is critical, as neurological damage may be fully reversible with timely treatment [5].

Notably, this case underscores that serum B12 levels in the low-normal range do not exclude deficiency. Several reports have highlighted the phenomenon of functional B12 deficiency, where intracellular cobalamin activity is insufficient despite seemingly adequate serum concentrations [9]. This may result from impaired transport, storage, or absorption, as seen in pernicious anemia. The presence of intrinsic factor and parietal cell antibodies in our patient confirms an autoimmune etiology affecting B12 uptake at the ileal level [1,3].

Although MMA and homocysteine testing were not available in this case, their inclusion is valuable in confirming functional B12 deficiency, particularly in patients with low-normal serum levels [7,8]. These biomarkers are more sensitive indicators of intracellular cobalamin deficiency and are recommended in recent guidelines [3].

MRI served as a valuable diagnostic and prognostic tool in this case. SCD classically manifests as bilateral, symmetric T2 hyperintensities in the posterior spinal cord, typically in the cervical region, which may completely resolve after B12 repletion [5,9]. The axial MRI image demonstrated the classic “inverted V sign,” representing bilateral dorsal column demyelination, commonly seen in SCD [4]. Several studies have emphasised that such radiological changes correlate with clinical status and may be reversible in early stages but become permanent with delayed intervention [1,10].

Furthermore, the absence of anemia, along with a normal neurological examination on presentation, aligns with previous findings, suggesting that neurological B12 deficiency can exist in the absence of classic hematological clues [2,8]. This highlights the importance of clinical vigilance, particularly in patients with risk factors such as autoimmune gastritis, vegan diets, prior gastric surgery, or long-term metformin use. Copper deficiency, though not tested in this patient, is a known mimicker of SCD and should be considered in the differential diagnosis of dorsal column myelopathy [11].

Hydroxocobalamin was selected for therapy due to its superior tissue retention and central nervous system penetration compared to methylcobalamin. It is the recommended formulation in cases of SCD [3].

Overall, this case highlights the importance of maintaining a high index of suspicion for B12 deficiency in patients presenting with posterior column symptoms, even in the absence of anemia or overt nutritional deficiency. Early treatment may prevent irreversible morbidity.

## Conclusions

This case highlights the importance of maintaining clinical suspicion for vitamin B12 deficiency even when initial laboratory findings are inconspicuous. Lhermitte’s sign, typically associated with demyelinating or compressive cervical pathology, may serve as an early and isolated manifestation of reversible metabolic myelopathy. The presence of neurological symptoms in the setting of low-normal serum B12 and positive intrinsic factor antibodies strongly supports the diagnosis of functional B12 deficiency. Early intervention with parenteral B12 can lead to complete neurological and radiologic recovery, as demonstrated in this case. Clinicians should consider B12 deficiency in the differential diagnosis of posterior column symptoms, especially in patients with risk factors for malabsorption, to prevent irreversible neurological damage through timely recognition and treatment.
